# Super-Refractory Status Epilepticus Treated with High Dose Perampanel: Case Series and Review of the Literature

**DOI:** 10.1155/2019/3218231

**Published:** 2019-09-02

**Authors:** Christopher R. Newey, Naresh Mullaguri, Stephen Hantus, Vineet Punia, Pravin George

**Affiliations:** ^1^Cerebrovascular Center, Neurological Institute, Cleveland Clinic, 9500 Euclid Avenue, Cleveland, OH 44195, USA; ^2^Epilepsy Center, Neurological Institute, Cleveland Clinic, 9500 Euclid Avenue, Cleveland, OH 44195, USA

## Abstract

**Introduction:**

Acute symptomatic seizures are frequent in the critically ill patient and can be difficult to treat. The novel anticonvulsant perampanel may be effective in the treatment of status epilepticus considering its mechanism of action of being an AMPA antagonist. We present four cases of super refractory status epilepticus treated with high dose perampanel.

**Method:**

Case report.

**Cases:**

Four patients were treated with perampanel for their refractory status epilepticus. One patient had new onset refractory status epilepticus of unknown etiology. Three other patients had status epilepticus as a result of their cardiac arrest. Two of the cardiac arrest patients had myoclonus. In all patients, the additional of perampanel resulted in a reduction of seizure burden without affecting hemodynamics or hepatic or renal function.

**Conclusion:**

Perampanel may be effective in the treatment of super-refractory status epilepticus of varying etiologies. A larger, prospective study is needed to further assess this therapy.

## 1. Introduction

Acute symptomatic seizures—convulsive or nonconvulsive— are frequent in the critically ill patient [1]. As seizures persist, the efficacy of GABAergic agents is reduced by the internalization of postsynaptic GABA-A receptors allowing for glutamate to promote ictal activity by binding to AMPA receptors [2]. Stepwise algorithms have been created to guide medical management with little to poor evidence behind them outside of administering benzodiazepine early [3–5]. Unfortunately, the treatment of status epilepticus can be difficult and is often associated with high morbidity [6–8]. Newer anticonvulsants, such as perampanel, may be effective and well-tolerated in the treatment of status epilepticus [9]. We present four cases of super refractory status epilepticus, in very different clinical settings, treated with high dose perampanel.

## 2. Cases

### 2.1. Case 1

A 28-year-old male with history of smoking and autism presented with acute altered mental status, increased combativeness, and an upper respiratory viral illness. He had no prior history of seizures or head trauma. Urine drug screen was positive for the tetrahydrocannabinol. His initial physical and neurological examinations were unremarkable. Labs were remarkable for leukocytosis (13040 cells/mm^3^) and mild transaminitis (alanine transaminase 102 U/mL and aspartate transaminase 75 U/mL). Hepatitis panel and human immunodeficiency virus serologies were unremarkable. Computerized tomography of the brain was unremarkable.

**Figure 1:**
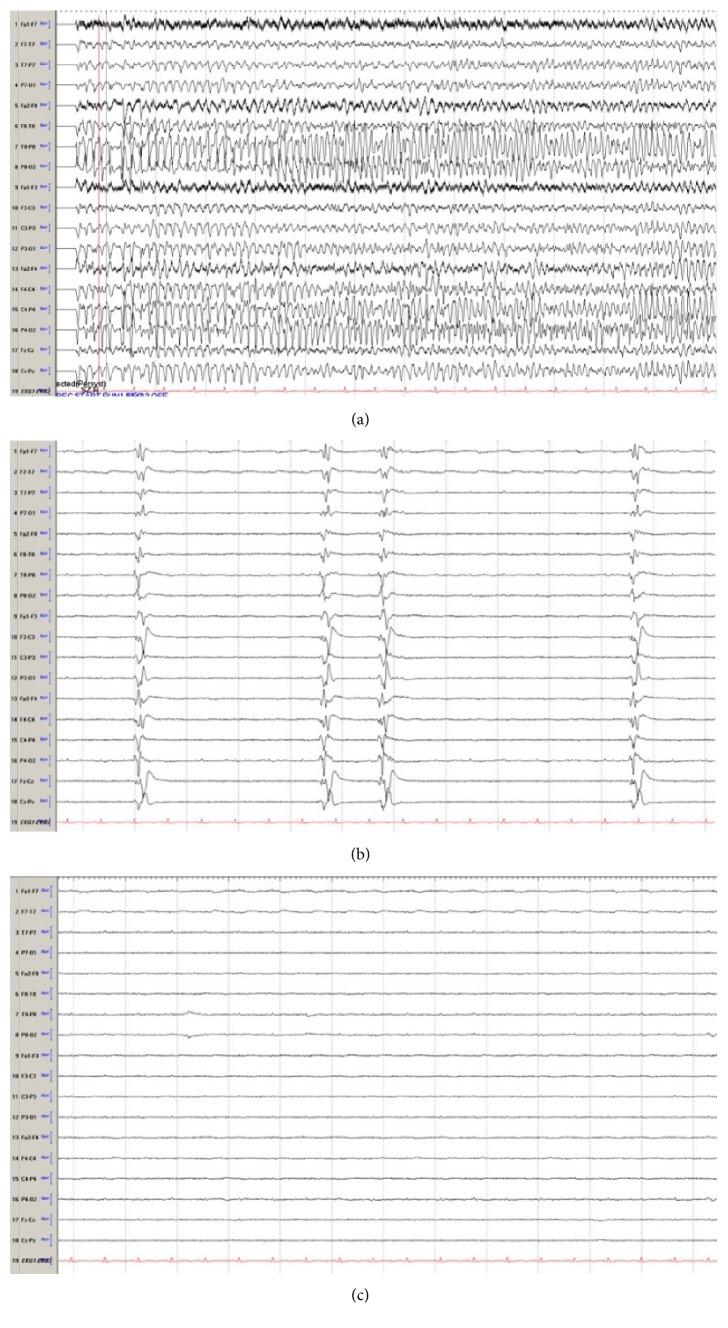
Electroencephalography (EEG): (a) EEG seizure arising from the right hemisphere, maximum temporo-parietal region. Seizures were seen occurring at a rate of 6–10 per hour lasting up to 3 minutes in duration without clinical signs. Seizures were frequent despite levetiracetam, lacosamide, phenobarbital, valproic acid, phenytoin, ketamine, midazolam, and pentobarbital. Sharply contoured generalized periodic discharges were seen interictally (b). Two hours after perampanel load (32 mg), the EEG transitioned to background suppression, and no further seizures were observed for the next 72 hours (c). Bitemporal montage, sens 7 uV, Lf 1.6 Hz, HF 70 Hz, 15 s epoch.

While in the emergency department (ED), he complained of severe thirst and suddenly suffered a generalized tonic-clonic seizure with urinary incontinence lasting approximately one-minute. He was treated with intravenous lorazepam and levetiracetam. He was admitted to the neurological intensive care unit. A lumbar puncture was performed which revealed a mildly elevated protein (84 mg/dL), lymphocytic predominant pleocytosis (16 cells/mm^3^), and normal glucose. He was started on broad-spectrum antibiotics including vancomycin, ceftriaxone, and acyclovir. VZV-PCR, and HSV-PCR returned negative; thus, antibiotics and anti-virals were de-escalated. Magnetic resonance imaging (MRI) of the brain with and without contrast was unremarkable. He was monitored on continuous electroencephalography (CEEG). Two days into his hospitalization, he had worsening exam and required intubation for airway protection. CEEG showed nonconvulsive seizures from bilateral frontotemporal regions. His levetiracetam dose was increased and he was given fosphenytoin. Despite these changes, his seizures progressed to convulsive and nonconvulsive status epilepticus. Autoimmune and paraneoplastic encephalitis panels were negative except for elevated anti-thyroid peroxidase antibodies (>1548 IU/mL (range 0–60 IU/mL)) and anti-thyroglobulin antibodies (128 IU/mL (range 0–60 IU/mL)). He was started on intravenous high dose methylprednisolone (1 g/day). Repeat lumbar puncture showed improving pleocytosis (13 cells/mm^3^) and protein (54 mg/dL). He continued to have nonconvulsive seizures on CEEG. He was started on continuous infusion of midazolam along with the propofol to achieve burst suppression. He was also loaded with phenobarbital. He continued to have breakthrough seizures and required pentobarbital. He was subsequently loaded with lacosamide and valproic acid and was eventually started on a ketamine infusion. Given lack of response to high dose steroids, plasma exchange was started on day ten of his hospitalization. He received 4 total doses of plasma exchange. His CEEG showed predominantly a burst suppression pattern but prolonged bursts (>30 s) had evolving seizures (Figure 1(a)). Thus, in an effort to break his continuous seizures, he was loaded with 32 mg of oral perampanel. A few hours after his perampanel dose, his continuous seizures broke, and he was able to be maintained in burst suppression to complete suppression for 48 hours with no clinical or electrographic seizures (Figures 1(b) and 1(c)). He was started on a maintenance perampanel dose of 8 mg daily. There were no hemodynamic or organ dysfunction from the perampanel load (Table 1).

Unfortunately, he developed an ileus with severe lactic acidosis and multiorgan failure. Family decided to pursue comfort measures. He passed away on day seventeen.

### 2.2. Case 2

**Figure 2:**
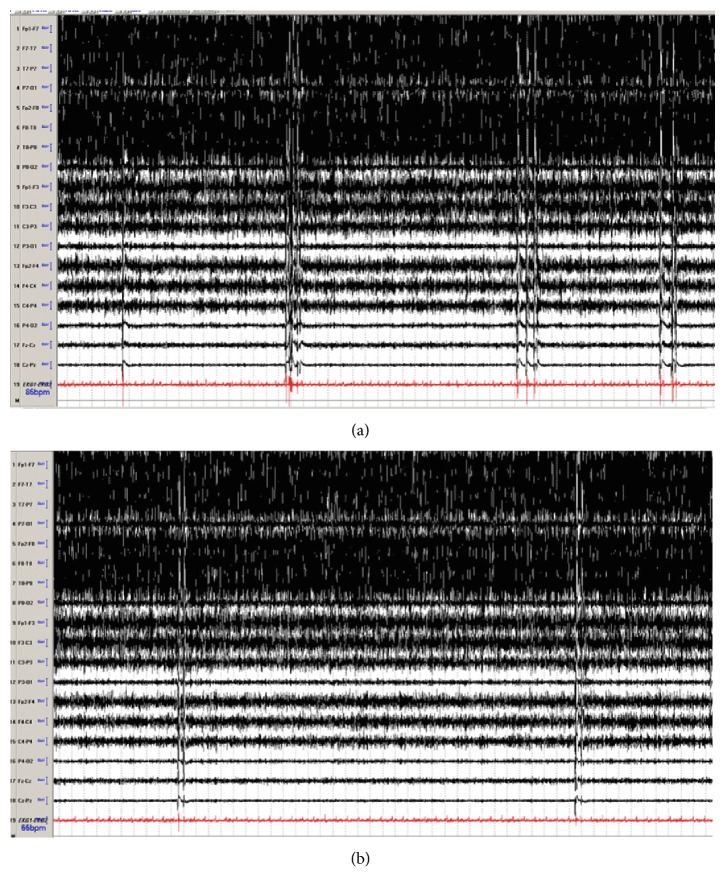
Electroencephalography (EEG): (a) Generalized burst suppression was seen occurring at a rate of 5–8 bursts per minute. Each burst was associated with generalized myoclonus despite the use of levetiracetam, valproic acid, and propofol. (b) Following 32 mg of perampanel, the bursts reduced to about 1-2 times per minute. Bitemporal montage, sens 7 uV, Lf 1.6 Hz, HF 70 Hz, 60 s epoch.

A 69-year-old man with no past medical history called emergency medical service (EMS) from home for new onset shortness of breath. On arrival to the house, there was no answer at the door. Forced entry was required. Patient was found on floor unresponsive in pulseless electrical activity (PEA). Advanced cardiac life support (ACLS) measures were started. He only required two rounds of chest compressions and epinephrine before return of spontaneous circulation (ROSC). He was taken to the ED where he was emergently intubated. CT of the head was negative for acute intracranial process. He was subsequently started on hypothermia protocol. He was admitted to the medical intensive care unit (MICU). On examination, he was noted to have myoclonic jerks particularly with stimulation. He was loaded with levetiracetam, valproic acid, and started on a propofol infusion. CEEG was placed which showed a burst-suppression pattern with 5–8 bursts per minute (Figure 2(a)). Each burst corresponded with myoclonus. His levetiracetam was adjusted without improvement. He was then loaded with 32 mg of oral perampanel through nasogastric tube. After 30 minutes, his CEEG showed 1-2 bursts per minute (Figure 2(b)). His bursts maintained this rate for next 8 hours. He had a mild transaminitis of 44 U/L ALT (alanine aminotransferase; normal 12–78 U/L) and 126 U/L AST (aspartate aminotransferase; normal 9–37 U/L) prior to anticonvulsants. Post anticonvulsants, his liver function tests and creatinine remained relatively unchanged (Table 1). His myoclonus improved and was not appreciated further after 36 hours from perampanel load. Two weeks after his admission, his neurological examination had no improved further. The family proceeded with comfort care measures.

### 2.3. Case 3

**Figure 3:**
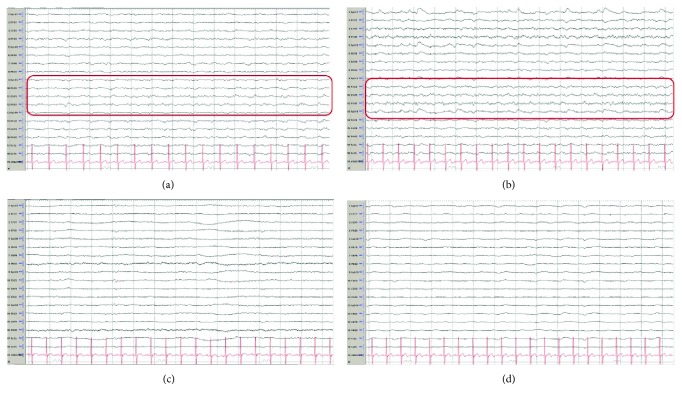
Electroencephalography (EEG): (a) Generalized slowing is seen with lateralized slowing in the left hemisphere. Periodic lateralized epileptiform discharges are seen in the left parieto-occipital region (red box). (b) Frequent seizures without clinical signs were seen arising from the left parieto-occipital region (red box). (c) Immediately before perampanel. At this time, the patient was receiving levetiracetam, lacosamide, valproic acid, and midazolam infusion. (d) 2 hours after perampanel, there is diffuse slowing. No further seizures occurred. Midazolam was stopped. He was eventually weaned off the levetiracetam and lacosamide and discharged on perampanel and valproic acid. Bitemporal montage, sens 7 uV, Lf 1.6 Hz, HF 70 Hz, 15 s epoch.

A 67-year-old man was admitted to an outside hospital (OSH) after being found unresponsive and in PEA. He received ACLS in the field. ROSC was obtained after 20 minutes. He was intubated and received induced hypothermia for 24 hours. He was rewarmed. His examination remained poor. On examination his pupils were sluggishly reactive. He had no motor response to noxious stimulation. His family requested transfer to our facility for second opinion. MRI of the brain was obtained which fluid attenuated inversion recovery changes in the bilateral basal ganglia. Somatosensory evoked potentials showed present N20 response bilaterally. CEEG showed generalized slowing as well as lateralized slowing in the left hemisphere with periodic discharges in the left parieto-occipital region (Figure 3(a)). He was started on levetiracetam. His CEEG then showed frequent non-convulsive seizures arising from the left parieto-occipital region (Figure 3(b)). He was started on lacosamide and then valproic acid with no improvement. Midazolam infusion was started along with load of perampanel 32 mg (Figures 3(c) and 3(d)). There was no change in his hepatic or renal panel, and his hemodynamics remained stable (Table 1). His maintenance dose was 12 mg for 5 days and then reduced to 6 mg. CEEG continued to be negative for seizures. His levetiracetam and lacosamide were weaned off. He was eventually discharged to long-term acute care hospital on valproic acid and perampanel 6 mg.

### 2.4. Case 4

**Figure 4:**
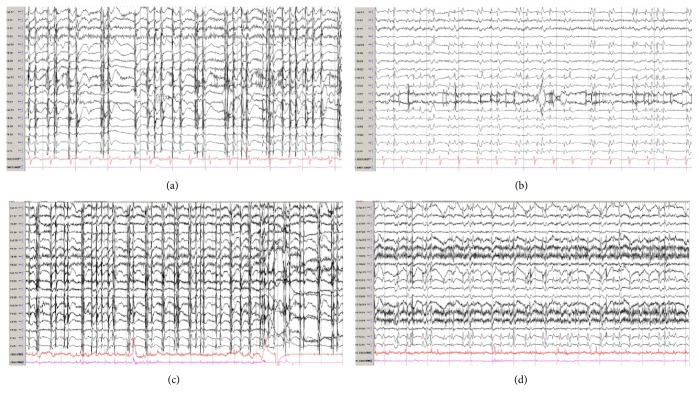
Electroencephalography (EEG): (a) Generalized myogenic artifact is seen throughout the record. (b) Following sedation and paralytic, generalized periodic discharges were seen. She was loaded with levetiracetam without improvement in the CEEG. (c) She was subsequently loaded with 32 mg of perampanel. (d) Two hours post load, her myoclonus had improved. CEEG, however, continued to show the generalized periodic discharge. Bitemporal montage, sens 7 uV, Lf 1.6 Hz, HF 70 Hz, 15 s epoch.

A 53-year-old woman with a history of congestive heart failure was admitted in PEA arrest post left heart catheterization. ROSC was obtained after 30 minutes. CT head was negative for any acute intracranial process. She underwent hypothermia (32C) for 24 hours followed by rewarming and normothermia. Her exam post rewarming showed intact brainstem reflexes and withdrawing in the extremities. Over the course of a week, she developed generalized myoclonic jerks, particularly of the axial muscles and face. It was unclear if these myogenic jerks had underlying CEEG correlate. She was subsequently given a sedative and a paralytic, which showed an underlying generalized periodic discharge (Figures 4(a) and 4(b)). She was loaded with levetiracetam (2000 g) without improvement in CEEG (Figure 4(c)). She was then loaded with 32 mg of perampanel. Two hours post perampanel load, her myogenic activity subsided, and her CEEG showed a generalized periodic pattern. She was maintained on 12 mg of perampanel daily for next three days. This was then reduced to 4 mg per day. Her myoclonus remained improved. There were no changes with her hepatic function panel or hemodynamics following the load of perampanel (Table 1). She eventually had a tracheostomy and PEG placed and transferred to a long-term acute care facility.

## 3. Discussion

These four cases highlight the effectiveness and tolerability of high dose perampanel in the management of patients with super refractory status epilepticus. Patient 1 had super refractory status epilepticus likely from an underlying autoimmune disorder. Patients 2, 3, and 4 had super refractory status epilepticus from varying degrees of anoxic injury. Patients 3 and 4 had generalized status epilepticus whereas patients 1 and 2 had focal seizures. In all cases, perampanel was effective in either stopping or reducing the seizure burden with no adverse effects.

Perampanel is a noncompetitive glutamate alpha-amino-3-hydroxy-5-methyl-4-isoxazolepropionic acid (AMPA) receptor antagonist [10]. It has a terminal half-life of ~105 hours and reaches steady state in 10–19 days [11]. It has a time to peak of 0.5 (fasting) to 2.5 hours (nonfasting) with 100% bioavailability and is extensively (>90%) metabolized by CYP450 3A4 [11]. Thus, a loading dose (such as 32 mg) may be necessary to achieve a therapeutic plasma level [2]. Additionally, a higher maintenance dose may be necessary to maintain the appropriate therapeutic serum concentration. Perampanel has been treatment of status epilepticus after failure of benzodiazepines [2, 12, 13]. This favorable profile is likely explained by the glutamate and gamma-aminobutyric acid (GABA) receptor composition in ongoing seizures [14, 15]. In a single-center study of 30 patients with refractory status epilepticus, the addition of perampanel was given following administration of standard treatment algorithm (median of 4 IV drugs, range 2–7 before perampanel) [13]. Perampanel was given as an initial higher dose (median 32 mg, range 16–32 mg) in 14 patients and a median of 4 mg (range 2–12 mg) in 16 patients [13]. Clinical response was observed by a median of 24 hours (range 8–48 hours) and EEG response by median of 60 hours (range 12–72 hours) with a shorter time to treatment response in those with high dose loading (16 vs 18 hours for clinical response; 24 vs 72 hours for EEG response) [13]. In ~23–63% of patients, a mild hepatic/cholestatic injury was observed following perampanel [10, 13]. No patient had changes in cardiorespiratory parameters following loading dose of perampanel [10, 13]. One patient developed multi-organ failure from the use of high doses of pentobarbital. We have previously reported on the observed surgical and medical complications that accompany high dose pentobarbital [6]. With the development of the new AEDs, the goal should be to stop seizures prior to having to use a barbiturate given the complications directly and indirectly associated with this therapeutic option.

Perampanel may also have a role in the treatment of post-anoxic status epilepticus. In a small pilot study of 8 post-anoxic patients with super-refractory, nonconvulsive status epilepticus (NCSE) and favorable prognostic indicators, the addition of perampanel (median dose of 6 mg; range 6–12 mg) after a median time of 9.5 days (range 4–35 days) from cardiac arrest to the standard of care [median number of 3 AEDs (range 2–5) and 3 anesthetics (range 1–4)] resulted in cessation of NCSE in six (75%) patients by 72 hours [10]. For those patients with postanoxic myoclonic seizures, perampanel has been shown to cause clinically meaningful improvement in myoclonus despite the use of other commonly administered antiepileptic medications [16]. These findings highlight the effectiveness of perampanel in refractory cases of status epilepticus.

These cases highlight the use of the novel anticonvulsant perampanel in the treatment of super-refractory status epilepticus. Perampanel was effective in treatment of status epilepticus in these cases with varying etiologies and was well tolerated. A larger, prospective study is needed to test this treatment option.

## Figures and Tables

**Table 1:** Hemodynamic and laboratory values for patients treated with high dose perampenal.

	Patient 1	Patient 2	Patient 3	Patient 4
*Systolic blood pressure (mmHg)*				
Baseline	109	144	111	98
2 hours post	106	142	101	101
24 hours post	102	135	129	91
48 hours post	105	106	133	92
*Creatinine (mg/dL)*				
Baseline	0.77	0.79	1.13	1.58
24 hours post	0.8	0.57	1.19	1.62
48 hours post	0.79	0.93	1.17	1.6
*Alanine aminotransferase (ALT) (U/L)*				
Baseline	137	41	77	85
24 hours post	53	44	47	79
48 hours post	56	26	26	68
*Aspartate aminotransferase (AST) (U/L)*				
Baseline	58	178	52	77
24 hours post	18	166	33	73
48 hours post	23	186	30	67

*Abbreviations:* mmHg, millimeters of Mercury; mg, milligrams; dL, deciliter; U, Units; L, liter.
